# Identification and characterization of plant-derived alkaloids, corydine and corydaline, as novel mu opioid receptor agonists

**DOI:** 10.1038/s41598-020-70493-1

**Published:** 2020-08-14

**Authors:** Teresa Kaserer, Theresa Steinacher, Roman Kainhofer, Filippo Erli, Sonja Sturm, Birgit Waltenberger, Daniela Schuster, Mariana Spetea

**Affiliations:** 1 Department of Pharmaceutical Chemistry, Institute of Pharmacy and Center for Molecular Biosciences Innsbruck (CMBI), University of Innsbruck, Innrain 80-82, 6020 Innsbruck, Austria; 2 Department of Pharmacognosy, Institute of Pharmacy and Center for Molecular Biosciences Innsbruck (CMBI), University of Innsbruck, Innrain 80-82, 6020 Innsbruck, Austria; 3 Department of Medicinal and Pharmaceutical Chemistry, Institute of Pharmacy, Paracelsus Medical University, Strubergasse 22, 5020 Salzburg, Austria

**Keywords:** Drug discovery, Medicinal chemistry, Pharmacology, Computational chemistry, Natural products, Pharmacology, Small molecules

## Abstract

Pain remains a key therapeutic area with intensive efforts directed toward finding effective and safer analgesics in light of the ongoing opioid crisis. Amongst the neurotransmitter systems involved in pain perception and modulation, the mu-opioid receptor (MOR), a G protein-coupled receptor, represents one of the most important targets for achieving effective pain relief. Most clinically used opioid analgesics are agonists to the MOR, but they can also cause severe side effects. Medicinal plants represent important sources of new drug candidates, with morphine and its semisynthetic analogues as well-known examples as analgesic drugs. In this study, combining in silico (pharmacophore-based virtual screening and docking) and pharmacological (in vitro binding and functional assays, and behavioral tests) approaches, we report on the discovery of two naturally occurring plant alkaloids, corydine and corydaline, as new MOR agonists that produce antinociceptive effects in mice after subcutaneous administration via a MOR-dependent mechanism. Furthermore, corydine and corydaline were identified as G protein-biased agonists to the MOR without inducing β-arrestin2 recruitment upon receptor activation. Thus, these new scaffolds represent valuable starting points for future chemical optimization towards the development of novel opioid analgesics, which may exhibit improved therapeutic profiles.

## Introduction

**Figure 1:**
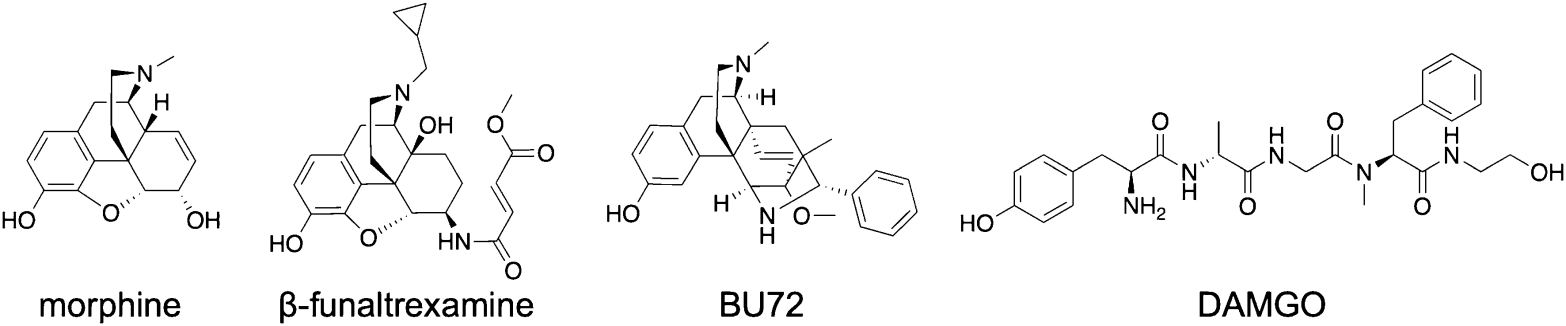
Chemical structures of morphine, β-funaltrexamine, BU72 and DAMGO.

Naturally occurring opioid alkaloids, such as morphine (Fig. 1), have been used for centuries for severe and chronic pain relief^1^. Over several decades, new opioids with diverse scaffolds were synthesized, pharmacologically evaluated and clinically used as the most effective class of analgesic drugs^2–5^. However, all currently available opioid analgesics share a similar spectrum of undesirable side effects, including respiratory depression, constipation, sedation, nausea and analgesic tolerance^5,6^. Additionally, the potential for addiction and abuse of opioids has seriously hindered their clinical application, with a huge rise in opioid misuse and overdose deaths resulting in an ongoing and rapidly emerging opioid epidemic worldwide^7,8^. Currently, intensive research focuses on finding new, innovative medications and technologies to treat opioid addiction, together with the discovery of safe, effective, non-addictive drugs to manage chronic pain^9–12^.

Opioids produce their pharmacological effects through the activation of opioid receptors, which include three main types, mu (MOR), delta (DOR) and kappa (KOR)^13,14^, of which the MOR type is the primary target of most clinically used opioid analgesics^3,5^. Opioid receptors share high homology and belong to the superfamily of seven transmembrane-spanning G protein-coupled receptors (GPCRs). Because of its therapeutic relevance, the MOR is among the few GPCRs determined in different activation states, with the first X-ray crystal structure of the receptor protein bound to β-funaltrexamine (Fig. 1), an irreversible antagonist (PDB entry 4DKL)^15^, and the 3D-structure in the active conformation where the receptor was co-crystallized with the agonist BU72 (Fig. 1) (PDB entry 5C1M)^16^. Recently, cryo-electron microscopy (cryo-EM) structures of the MOR (PDB entries 6DDE and 6DDF) bound to the agonist peptide DAMGO (Fig. 1) were reported^17^.

The detailed structural information of the MOR available nowadays^15–17^, as well as the emerging concept of biased agonism to the MOR^5,12,18^, provide innovative research directions that not only aid to understand MOR-mediated signaling and its pharmacology, but also offer novel opportunities for the discovery of new opioid therapeutics^19^. Further, an important source of new drug candidates is represented by natural product medicines, having a long history of use in the treatment and prevention of many human diseases^20,21^. Natural products and their derivatives account for about half of approved drugs^20^. Morphine (Fig. 1), the structure on which the vast majority of semisynthetic opioids (e.g. oxycodone, oxymorphone and hydromorphone) is based, is an alkaloid found in the poppy plant, *Papaver somniferum*^2,3^. Other recent examples are the indole alkaloid mitragynine isolated from *Mitragyna speciosa*, known as “kratom”, and its active metabolite 7-hydroxymitragynine, that are viewed as potential analgesic drugs^22–24^.

**Figure 2:**
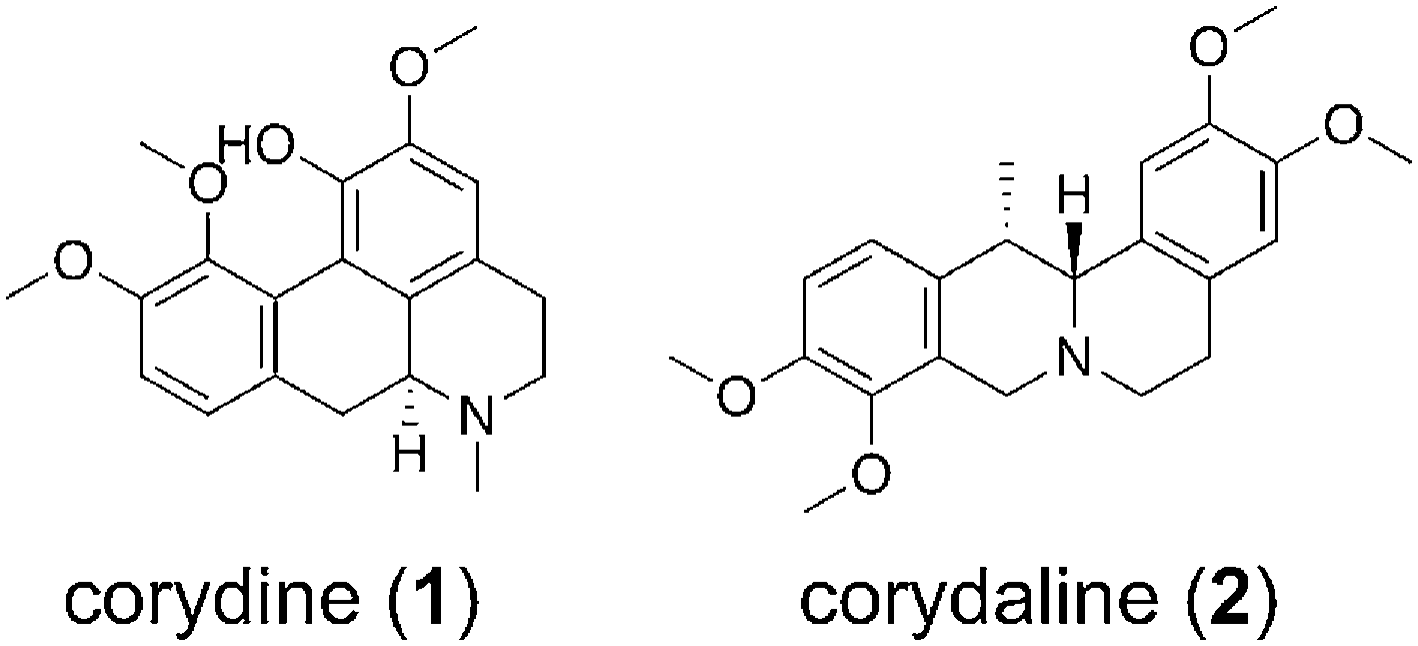
Chemical structures of corydine (**1**) and corydaline (**2**).

In the search for ligands with new chemotypes and further understanding the mechanism by which known ligands (i.e. small molecules and peptides) bind and activate the MOR, structure-based discovery campaigns have used the high-resolution MOR structures to computationally investigate diverse molecules^25–31^. We have previously reported on a virtual screening campaign that led to the identification of novel chemotypes that displayed MOR antagonism in vitro and in vivo^26^. In this study, we generated a collection of virtual screening protocols based on different in silico methods, such as pharmacophore- and shape-based modelling and docking. After theoretical validation, we prospectively applied these protocols to a library of synthetic compounds, and the MOR activity of three virtual screening hits could be confirmed experimentally. Structural analogues of one of these validated hits were reported as natural products isolated from different *Berberis* species^26^. This prompted us to apply the computational models to an in-house library containing, beyond others, also *Berberis* constituents. In the present study, by combining molecular modeling and pharmacological approaches, we report on the discovery of two plant-derived alkaloids, corydine (**1**) and corydaline (**2**) (Fig. 2), as new MOR agonists with a G protein-biased profile.

## Results

### Molecular modeling and virtual screening

**Figure 3:**
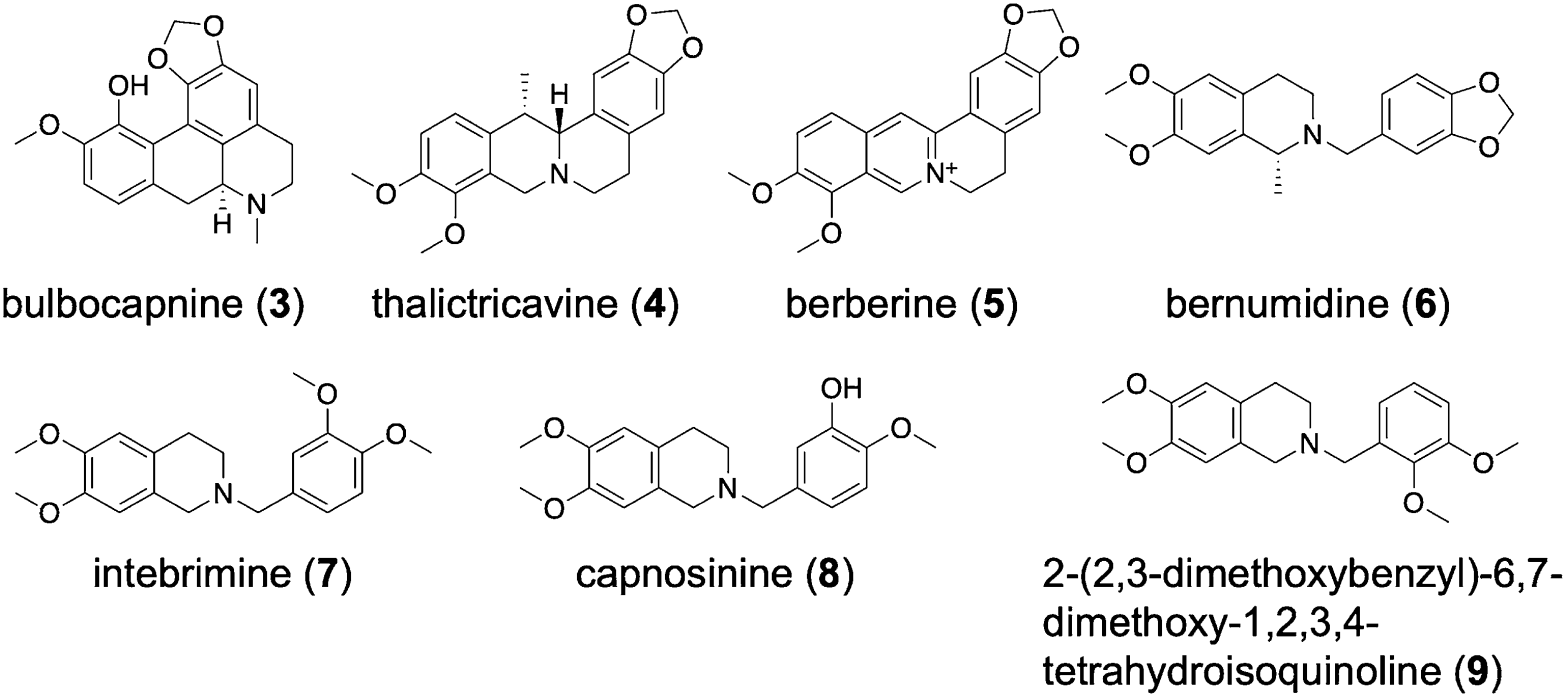
Chemical structures of compounds **3**–**9**.

**Figure 4:**
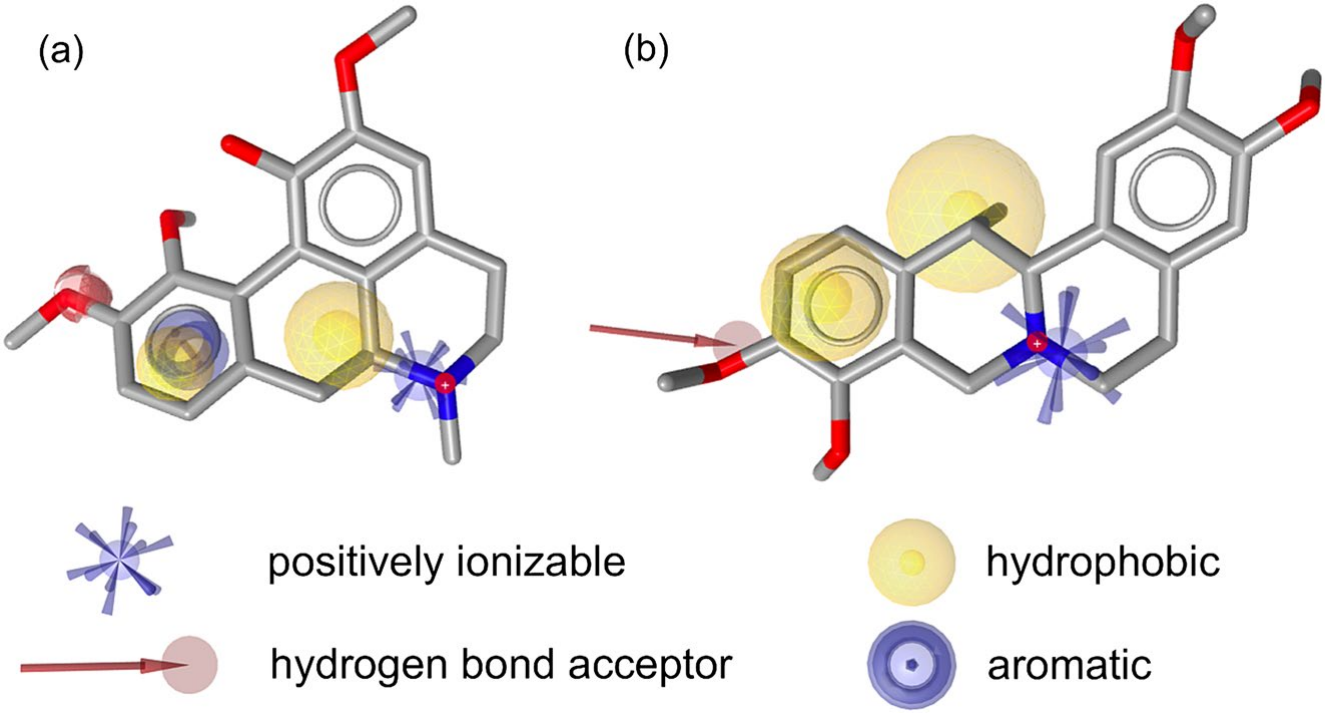
Exemplary virtual hit compounds mapping MOR agonist pharmacophore models. (**a**) Corydine (**1**) mapped into model pm-ag-lig-model-1, and (**b**) corydaline (**2**) mapped model pm-ag-4dkl-model-13.

We have previously reported the generation, validation, and prospective application of a set of MOR agonist and antagonist pharmacophore models^26^. Whereas the three agonist models mapped mainly MOR agonists during theoretical validation, the antagonist models proved to have little discriminative power (i.e. agonists vs. antagonists) and should therefore rather be considered as general MOR ligand models. In this study, we have used this set of MOR agonist and antagonist pharmacophore models to screen a small in-house library of naturally occurring alkaloids and synthetic analogues. As none of the molecules matched any of the models when mapping of all features was required, the number of omitted features was increased to one, which means that compounds are also recognized as potentially active compounds if they miss one of the model features. Using these settings, we retrieved 15 virtual hits. The central role of Asp147 and Tyr148 of peptides, morphinans ligands, and other chemotypes for binding to the MOR is well recognized^22,24–34^. Further, both of these interactions appeared to be critical for ligand binding in our previous study^26^. Mapping of these features was therefore chosen as requirement for virtual hits in order to be subjected to experimental testing. Based on the current results, we have selected seven natural products, corydine (**1**), corydaline (**2**), bulbocapnine (**3**), thalictricavine (**4**), bernumidine (**6**), intebrimine (**7**) and capnosinine (**8**), and one natural product analogue, 2-(2,3-dimethoxybenzyl)-6,7-dimethoxy-1,2,3,4-tetrahydroisoquinoline (**9**) (Figs. 2 and 3) for further investigations. They were mapped by one (compounds **2**–**4** and **6**), two (compounds **1**, **7**, and **9**) or even three pharmacophore models (compound **8**) (Table 1). Figure 4 shows corydine (**1**) and corydaline (**2**) aligned to the pharmacophore models pm-ag-lig-model-1 and pm-ag-4dkl-model-13, respectively. Due to the structural similarity, berberine (**5**) was also added to the list of test compounds.

**Table 1:** MOR pharmacophore models mapping selected test compounds **1–9**.

Compound	Matching pharmacophore models^a^
Corydine (**1**)	pm-ag-lig-model-1, pm-ant-lig-model-4
Corydaline (**2**)	pm-ag-4dkl-model-13
Bulbocapnine (**3**)	pm-ant-lig-model-4
Thalictricavine (**4**)	pm-ag-lig-model-1
Berberine (**5**)	–^b^
Bernumidine (**6**)	pm-ant-lig-model-4
Intebrimine (**7**)	pm-ag-4dkl-model-13, pm-ant-lig-model-3
Capnosinine (**8**)	pm-ag-4dkl-model-13, pm-ant-lig-model-3, pm-ant-lig-model-4
**9**	pm-ag-lig-model-2, pm-ant-lig-model-3

^a^For details on the applied pharmacophore models, see Kaserer et al*.*^26^.

^b^Berberine (**5**) did not match a pharmacophore model, but was included in experimental testing due to structural similarity.

### Biological evaluation

Out of the nine compounds selected for biological testing, eight (compounds **1**–**8**) are natural products, whereas **9** is a synthetic compound, structurally related to the natural product intebrimine (**7**) (Figs. 2 and 3). A synopsis of their origin and known bioactivities is presented in the Supporting Information.

#### In vitro binding and activity to the MOR

**Figure 5:**
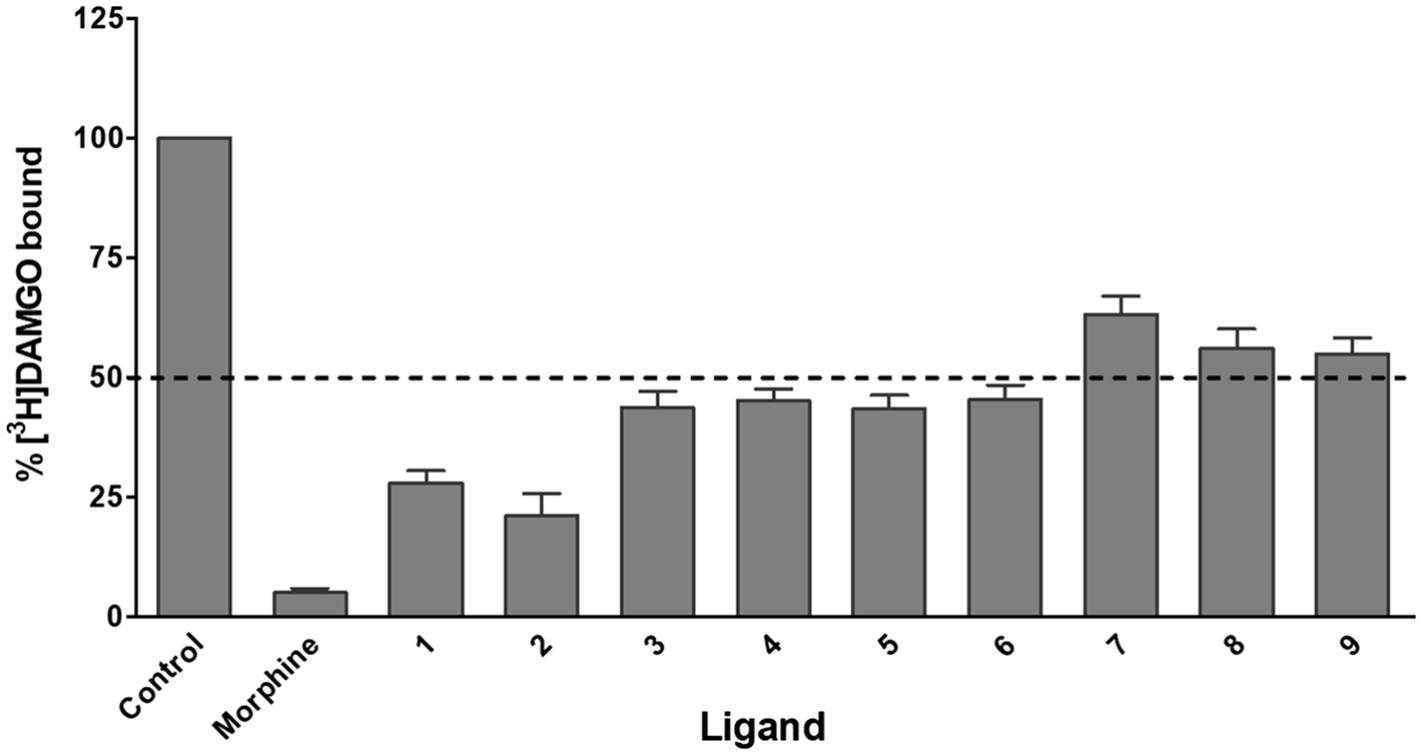
Competitive inhibition of [^3^H]DAMGO binding by compounds **1**–**9** to the human MOR**.** Membranes of CHO cells stably expressing the human MOR were incubated with [^3^H]DAMGO in the absence (control) or presence of compounds **1**–**9** (all 10 µM), or the reference MOR ligand morphine (10 µM). Values are means ± SEM (n = 3–4 independent experiments performed in duplicate).

**Figure 6:**
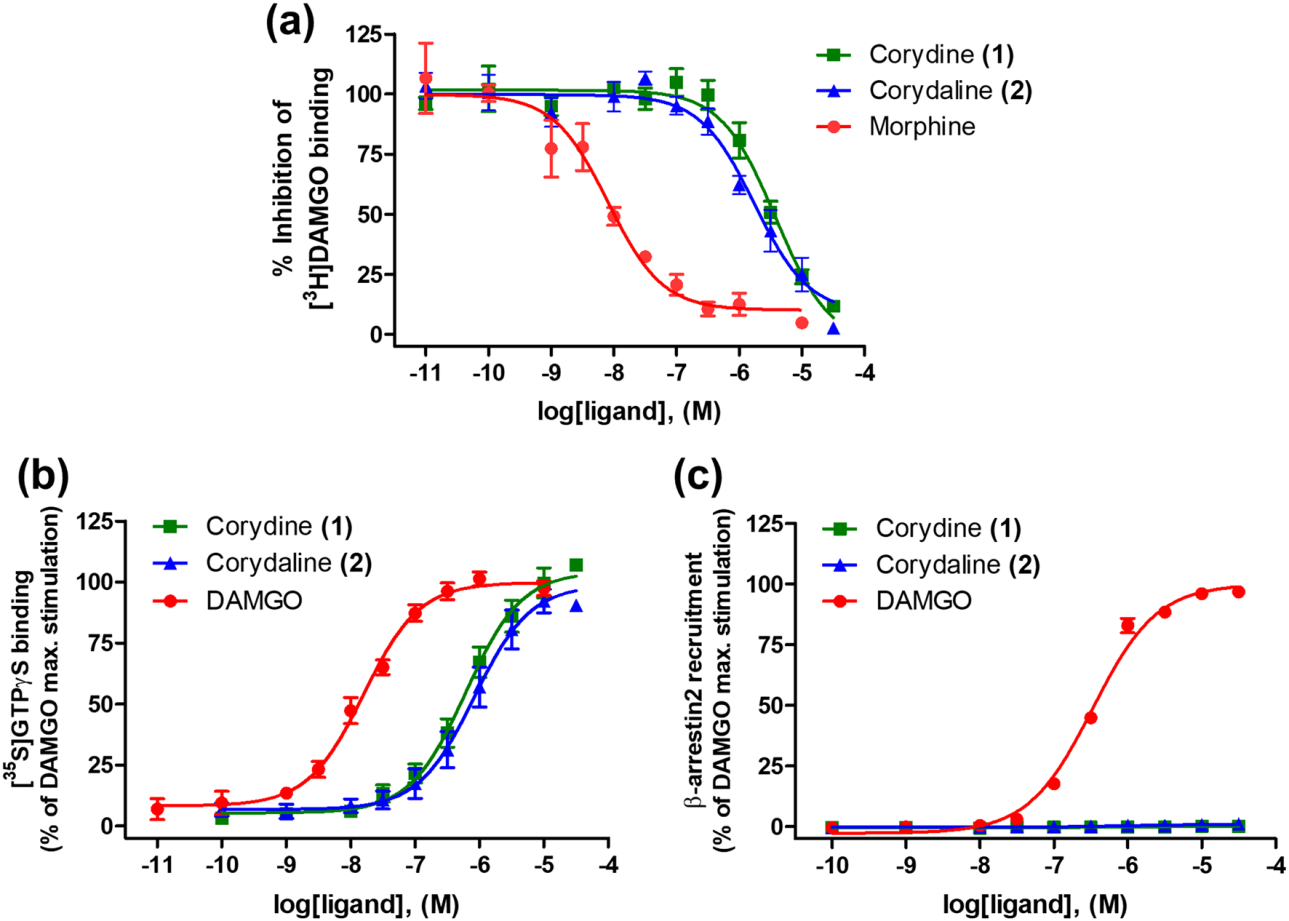
In vitro activity profiles of corydine (**1**) and corydaline (**2**) to the human MOR. (**a**) Concentration-dependent inhibition of [^3^H]DAMGO binding to CHO-hMOR cell membranes was determined in the competitive radioligand binding assay. (**b**) Agonist activities of test compounds to the MOR as stimulation of [^35^S]GTPγS binding were determined in the [^35^S]GTPγS binding assay with CHO-hMOR cell membranes. (**c**) β-Arrestin2 recruitment activities of test compounds to the MOR were determined in the PathHunter β-arrestin2 assay. Values are means ± SEM (n = 3–4 independent experiments performed in duplicate).

The initial biological screening was performed using a competitive radioligand binding assay to the human MOR with the eight natural products (**1**–**8**) and the synthetic compound **9**. The ability of the nine compounds and the reference MOR ligand, morphine, to inhibit binding of the selective MOR radioligand [^3^H]DAMGO was assessed with membranes from Chinese hamster ovary cells stably expressing the human MOR (CHO-hMOR), according to previously described procedures^26^. Natural products **1**–**6** inhibited [^3^H]DAMGO binding to the MOR by > 50% (Fig. 5). Of these, corydine (**1**) and corydaline (**2**) showed to be most potent in competing with [^3^H]DAMGO for binding to the MOR. Therefore, they were selected for further investigations of their MOR activities. Both compounds produced concentration-dependent inhibition of [^3^H]DAMGO binding, displaying moderate binding affinities to the human MOR (Fig. 6a). Corydaline (**2**) displayed a binding affinity (as K_i_ value) to the MOR about 2-times higher than that on corydine (**1**), although it was much lower than the MOR affinity of morphine (Table 2). Additional in vitro binding studies established that corydine (**1**) and corydaline (**2**) did not specifically bind to the human DOR and KOR expressed in CHO cells (Figure S1 and Table S1).

**Table 2:** In vitro binding affinities of corydine (**1**) and corydaline (**2**) to the MOR.

Compound	K_i_ (µM)^a^
Corydine (**1**)	2.82 ± 0.61*
Corydaline (**2**)	1.23 ± 0.29
Morphine	0.0067 ± 0.0009

^a^Determined in competitive radioligand binding assays using CHO-hMOR cell membranes. Inhibitory constant (K_i_) values were calculated from the competition binding curves by nonlinear regression analysis. Morphine was used as reference MOR ligand. Values are means ± SEM (n = 3–4 independent experiments performed in duplicate). *P < 0.05 for corydine (**1**) vs. corydaline (**2**) (unpaired *t*-test).

To evaluate whether corydine (**1**) and corydaline (**2**) behave as agonists or antagonists to the MOR, we used the in vitro [^35^S]GTPγS functional assay, which measures MOR-mediated G protein activation. The assay was performed according to earlier described procedures^26^. As shown in Fig. 6b, both ligands produced a concentration-dependent increase in the [^35^S]GTPγS binding in CHO-hMOR cell membranes, having high efficacy and acting as full agonists to the human MOR. Corydine (**1**) displayed about 3-times greater potency compared to corydaline (**2**), while being 34-times less potent as an agonist than the reference MOR agonist DAMGO (Table 3).

**Table 3:** In vitro functional activities of corydine (**1**) and corydaline (**2**) to the MOR.

Compound	G protein activation^a^		β-arrestin2 recruitment^b^	
	EC_50_ (µM)	%stim	EC_50_ (µM)	%stim
Corydine (**1**)	0.51 ± 0.11*	102 ± 6	–^c^	–^c^
Corydaline (**2**)	1.50 ± 0.44	104 ± 6	–^c^	–^c^
DAMGO	0.015 ± 0.002	100	0.34 ± 0.02	100

^a^Determined in the [^35^S]GTPγS binding assay using CHO-hMOR cell membranes.

^b^Determined in the PathHunter β‐arrestin2 recruitment assay with U2OS cells co‐expressing the hMOR and the enzyme acceptor tagged β‐arrestin2 fusion protein. Efficacies are expressed as percentage stimulation (% stim.) relative to DAMGO (reference MOR agonist).

^c^–denotes no measurable activity. Values are means ± SEM (n = 3–4 independent experiments performed in duplicate). *P < 0.05 for corydine (**1**) vs. corydaline (**2**) (unpaired *t*-test).

As GPCRs, MOR can activate parallel or distinct signaling pathways in addition to G protein signaling, the principle among them being the β-arrestin2-dependent signaling^5,12,18^. While the G protein-mediated signaling is linked to beneficial effects (i.e. analgesia), the β-arrestin2 signaling pathway appears to be responsible for the undesirable effects (i.e. respiratory depression, constipation, tolerance and dependence) of MOR agonists. The concept of biased agonism or functional selectivity was introduced as a means to separate desirable and adverse drug responses^8,35^, and the in vivo relevance of this phenomenon has attained much attention in the past years^18,36^. Targeting biased agonism to the MOR has gained significance for drug discovery over the recent years, where G protein-biased MOR agonists may deliver the desired analgesia without liability for unwanted side effects^27,36–39^. On this basis, we examined the capability of corydine (**1**) and corydaline (**2**) to promote MOR-mediated β-arrestin2 signaling in the PathHunter β-arrestin2 recruitment assay using U2OS cells co-expressing the human MOR and the enzyme acceptor tagged β-arrestin2 fusion protein^40^. In this functional assay, test compounds were examined in parallel with DAMGO, which served as the reference MOR agonist. Interestingly, corydine (**1**) and corydaline (**2**) failed to induce β-arrestin2 recruitment upon activation of the MOR, whereas DAMGO effectively recruited β-arrestin2 (Fig. 6c, Table 3). Since both compounds **1** and **2** exhibit significant efficacy for G protein activation in the [^35^S]GTPγS binding assay (Fig. 6b), there is a strong bias in favor of G protein signaling. β-Arrestin2 recruitment was too low in the range of tested concentrations to permit a formal determination of a bias factor, which essentially defines the extent of differences in relative agonist activity between two assays^41^.

#### Antinociceptive activity

**Figure 7:**
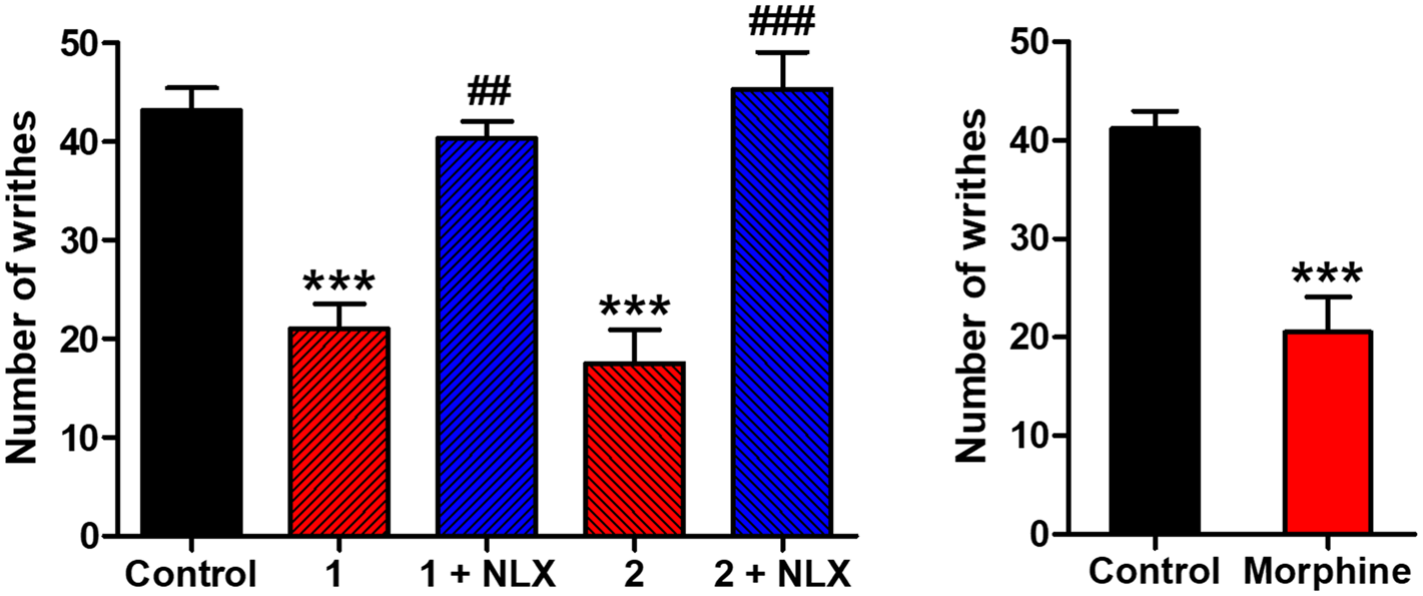
Antinociceptive effects of corydine (**1**), corydaline (**2**), and the reference MOR ligand morphine in the acetic acid-induced writhing assay in mice after s.c. administration**.** Mice received s.c. vehicle (control), test compounds, or morphine, and the number of writhes were counted at 30 min after administration of compounds **1** (5 mg/kg) and **2** (10 mg/kg), or morphine (0.5 mg/kg) administration, for a period of 10 min. Naltrexone (NLX, 1 mg/kg) was s.c. administered 10 min before compounds **1** or **2**. Values are means ± SEM (n = 5–6 mice per group). **P < 0.01 and ***P < 0.001 vs. control group; ^##^*P* < 0.01 vs. agonist-treated group; one-way ANOVA followed by Tukey’s post hoc test or unpaired *t*-test.

The MOR agonist activity of corydine (**1**) and corydaline (**2**) was further evaluated in vivo in a mouse model of chemical sensitivity, the writhing assay, a widely used model of visceral pain^42^. This test involves intraperitoneal (i.p.) injection of acetic acid, which results in abdominal constriction, causing the mice to writhe^26^. Both compounds showed antinociceptive effects in mice after subcutaneous (s.c.) administration by significantly inhibiting the writhing behavior (Fig. 7). Corydine (**1**) and corydaline (**2**) administered at 5 and 10 mg/kg, respectively, caused a significant reduction in the number of writhes by 51% and 59%, respectively. At tested doses, no alterations in animal’s general behavior (i.e. sedation and motor impairment) were observed. Compared to morphine (0.5 mg/kg), tested at equianalgesic doses, **1** and **2** were ca. 10- and 20-times, respectively, less effective. The antiwrithing response of corydine (**1**) and corydaline (**2**) was antagonized by the MOR antagonist naltrexone, demonstrating a MOR-mediate mechanism of action (Fig. 7).

### Binding hypotheses for corydine (1) and corydaline (2) to the MOR

**Figure 8:**
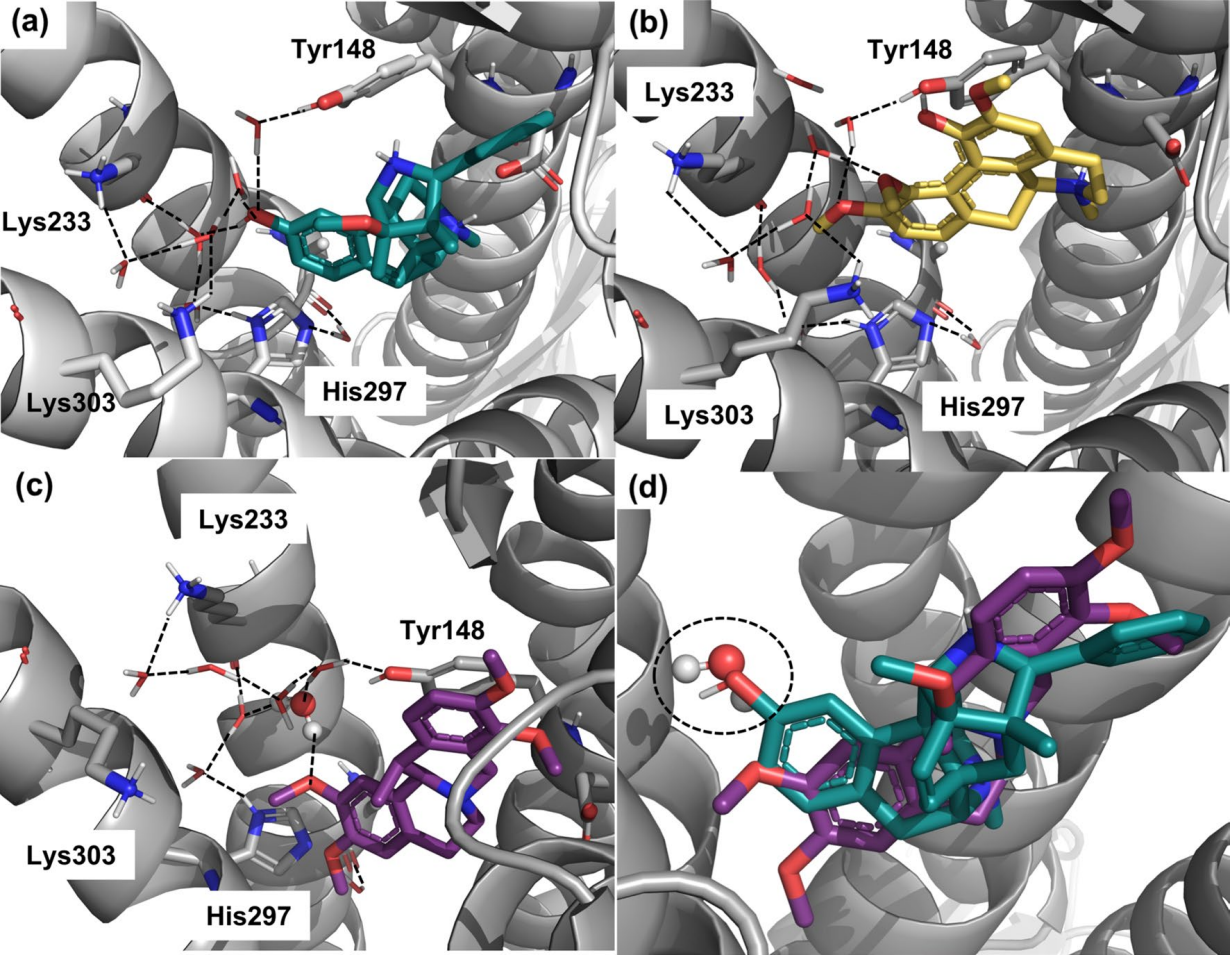
Predicted binding modes of corydine (**1**) and corydaline (**2**) to the MOR. (**a**) The crystal structure of MOR (gray) in complex with the agonist BU72 (green sticks, PDB entry 5C1M) reveals that the phenolic OH group is involved in an intricate water network (dashed lines) connecting the compound to Tyr148, His297, Lys233, and Lys303. (**b**) Induced fit docking of corydine (**1**) (yellow sticks) suggests that this water network is largely maintained upon corydine binding, although the absolute position of water molecules had to be adapted due to the methoxy-groups. (**c**) In the induced fit docking pose, corydaline (**2**) (violet sticks) requires an additional water molecule (highlighted as spheres) to maintain the water network. In addition, interaction with Lys303 is lost. (**d**) This additional water molecule overlays with the OH-group of BU72 (highlighted with dashed lines).

Experimentally, we established that corydine (**1**) displays a more potent MOR activation despite lower binding affinity compared to corydaline (**2**) (Tables 2 and 3). Although the differences in affinity (K_i_) and potency (EC_50_) values between the two compounds seem minor, we found them to be statistically significant (*P* < 0.05, unpaired *t*-test). In our experience, corydine (**1**) shows a highly unusual profile. To investigate whether we could find a potential structural explanation for this observation, we have generated in silico binding models to the MOR for the two compounds. The crystal structure of BU72 in complex with murine MOR (PDB entry 5C1M)^16^ reveals an intricate water network, that connects the ligand with the residues Lys233, Lys303, His297 and Tyr148 (Fig. 8a). As these water molecules appear to have a functional role, we included them in the structural modelling. Both corydine (**1**) and corydaline (**2**) (Fig. 2) are structurally distinct from the morphinan agonist BU72 (Fig. 1). To account for these differences and allow for some structural adaptions, we decided to employ the induced fit docking procedure in Maestro^43^. Analysis of the proposed binding modes suggests that the observed differences in receptor activation could be due to alterations of the water network mediating interactions between the agonists and the MOR in the active conformation. In our model, corydine (**1**) is involved in a similar water network as BU72 (Fig. 8b), explaining the lower EC_50_ value at the MOR (i.e. more potent activation) compared to corydaline (**2**) despite weaker binding affinity to the MOR (Tables 2 and 3). On the other hand, corydaline (**2**) may require an additional water molecule to maintain this water network (Fig. 8c), potentially rendering it less potency in activating the receptor. Notably, this additional water molecule occupies a similar position as the BU72-OH group in the crystal structure (Fig. 8d).

### In silico profiling of off-target activity and metabolites of corydine (1) and corydaline (2)

To investigate potential off-target effects of corydine (**1**) and corydaline (**2**), the compounds were subjected to in silico target profiling using the Similarity Ensemble Approach (SEA) (https://sea.bkslab.org/)^44^. Among other targets, multiple dopamine receptor subtypes were predicted, in line with previous reports by Ma et al.^45^ and Wu et al.^46^ A detailed summary of in silico off-target prediction results is provided in Table S1. Furthermore, to rule out that the observed in vivo effects were mediated by potential active metabolites, we subjected metabolites of corydaline (**2**) (**16**–**22**) described in Ji et al*.*^47^ as well as predicted metabolites of corydine (**1**) (**10**–**15**) (Figure S2) to in silico target profiling using SEA. Similar to the parent compounds, metabolites were also projected to have activity at the dopamine receptors along other targets. A detailed summary of investigated metabolites and predicted targets is presented in Figure S2 and Table S3. Noteworthy, MOR was not suggested as target for neither parent compounds nor metabolites, indicating that corydine (**1**) and corydaline (**2**) indeed represent novel chemical scaffolds to this receptor.

## Discussion and conclusions

In the present study, combining in silico (pharmacophore-based virtual screening and docking) and pharmacological (in vitro and in vivo assays) approaches, we report on the discovery of two natural products, corydine (**1**) and corydaline (**2**), as new MOR agonists that produce antinociceptive effects in mice after s.c administration via a MOR-dependent mechanism. Furthermore, corydine (**1**) and corydaline (**2**) were identified as G protein-biased agonists to the MOR without inducing β-arrestin2 recruitment. This phenomenon, known as ‘functional selectivity’ or ‘biased agonism’, has gained increased attention to GPCR drug discovery towards effective and safer therapeutics, including opioid analgesics^12,18,48^.

Among the neurotransmitter systems involved in pain perception and modulation, the opioid system, particularly the MOR, is one of the most important^5^. Most clinically available opioid analgesics are agonists to the MOR that are highly effective in relieving pain, but they also have severe side effects, including abuse and misuse liability^5–8^. Medicinal plants are tremendous sources of new drug candidates^20,21^. During the past decades, there has been a renewed interest in natural product research due to the drawback of alternative drug discovery methods to deliver lead compounds in key therapeutic areas, such as pain. Natural products are a robust source of unique structural scaffolds. The study of psychoactive natural products had a continuous influence on the understanding of their function in the central nervous system^23,49,50^. Notable examples are the alkaloid morphine from *Papaver somniferum* and Δ^9^-tetrahydrocannabinol (THC) from *Cannabis sativa* that led to the discovery of the endogenous opioid and endocannabinoid systems, respectively^13,14,51^. Evidence on the neuropsychiatric effects of natural agonists to the KOR in humans comes from experience with salvinorin A, the main active psychotropic molecule in *Salvia divinorum*^52^. Chemical derivatization and modification of psychoactive natural products have provided and continues to offer innovative scientific and therapeutic discoveries. The progress in medicinal chemistry, drug discovery technologies and significant advances in structural biology of GPCRs by means of modern methodological and powerful computational systems^26,27,49,52^ plays an essential role in such discoveries.

We have previously reported the generation, validation, and application of a set of MOR agonist and antagonist pharmacophore models^26^. By using this collection of models for pharmacophore-based virtual screening, corydine (**1**) and corydaline (**2**) were identified as active ligands to the MOR, albeit they interact with the MOR relatively weakly. It is commonly recognized that hits identified in a virtual screening campaign often display weaker activity than the compounds the models were based on^26,53^. Our docking study to the MOR revealed that both compounds share several essential receptor-ligand interactions, including the salt bridge with Asp147 and hydrogen bond formation with Tyr148 (water-mediated in the case of corydaline (**2**), analogous to BU72), as two residues recognized as key interaction sites for ligand (small molecules and peptides) binding to the MOR^24–34,54^. However, there are also receptor-ligand interaction pattern dissimilarities. The experimental differences in receptor activation, with corydine (**1**) being more potent than corydaline (**2**), are possibly due to alterations of the water network mediating interactions between the agonists and the MOR in the active conformation. Corydaline (**2**) may require an additional water molecule to maintain the water network, and we hypothesize that it is therefore less potent in activating the receptor.

Corydine (**1**) and corydaline (**2**) are two naturally occurring alkaloids in different *Corydalis* and *Berberis* species^45,55–60^ that are used as medicinal plants to treat pain (spastic pain, abdominal pain, or pain due to injury) and other human ailments^61–64^. Plant extracts and isolated alkaloids, mostly corydaline (**2**), were reported to produce antinociceptive effects in rodents^59,65–68^, although no mechanism of action was associated to the observed pain inhibitory effects so far. Further, pharmacokinetic studies demonstrated that corydaline (**2**) can effectively cross the blood–brain barrier in rats^69^, with *O*-demethylation and hydroxylation as the major metabolic pathways in human liver^47^. In vitro investigations, using cell-based functional assays, established corydaline (**2**) to bind to the dopamine D1 receptor with antagonist activity^45,46^, whereas no molecular target was attributed to the biological effects of corydine (**1**). Our in silico profiling study also revealed that multiple dopamine receptor subtypes have been prioritized by SEA^44^ for both compounds as well as for metabolities of corydine (**1**) and corydaline (**2**).

In the present study, we show that corydine (**1**) and corydaline (**2**) bind to the MOR and are full agonists to the receptor, and produce MOR-mediated antinociceptive effects in a mouse model of visceral pain (writhing assay) after s.c. administration. Besides analgesia, MOR agonists are well-known to induce other physiological and behavioral responses^5,6^. While generally, no major alterations in locomotor activity and no sedation were observed in animals at the tested doses of corydine (**1**) and corydaline (**2**), studies on side effects profiling may be of future interest. We also showed corydine (**1**) and corydaline (**2**) as G protein-biased agonists at the MOR, as they do not promote β-arrestin2 recruitment following receptor activation. Altogether, our findings indicate that the applied MOR pharmacophore models and virtual screening workflows have a clear potential for the discovery of novel bioactive molecules to the MOR. The new chemotypes, corydine (**1**) and corydaline (**2**) as natural products, showed MOR biased agonist properties, thus representing valuable starting points for further chemical optimization toward the development of novel opioid analgesics with potentially reduced side effects.

## Materials and methods

### In silico methods***. ***Virtual screening

A conformational database was generated for the in house compounds using Omega 2.5.1.4^70,71^ implemented in LigandScout 3.1^72,73^. A maximum number of 500 conformers were calculated per molecule. For pharmacophore based virtual screening with LigandScout 3.1, the MOR pharmacophore collection reported in Kaserer et al*.*^26^ was employed. Default settings were used except that the maximum number of omitted features was increased to 1.

### Induced fit docking

The crystal structure of the BU72-murine MOR complex (PDB entry 5C1M)^16^ was used to generate binding hypotheses for corydine (**1**) and corydaline (**2**). A longer stretch of the N-terminus is resolved in this structure compared to e.g. the MOR-β-funaltrexamine complex (PDB entry 4DKL)^15^ due to its involvement in BU72 binding. This N-terminal section is unlikely to participate in binding of the structurally unrelated alkaloids **1** and **2**, but may render parts of the binding site inaccessible. Therefore, residues 52–63 were deleted. All water molecules except 502, 505, 521, 526, 538, 553, 563, and 565 were removed. The structure was then prepared with the Protein Preparation Wizard^74^ in Maestro release 2019–4^43^. Briefly, bond orders were assigned, hydrogens added, selenomethionines were converted to methionines, missing side chains and loops were added, termini were capped, het states were generated, H-bonds assignment was refined, and a restrained minimization was conducted. The prepared structure was then used for induced fit docking of BU72 as a control, and corydine (**1**) and corydaline (**2**). The co-crystallized ligand BU72 was used to define the docking site and the default induced fit docking settings were applied.

### In silico profiling

The Smiles codes of corydine (**1**) and corydaline (**2**) were submitted to the Similarity Ensemble Approach (SEA) webserver (https://sea.bkslab.org/)^44^ for in silico profiling and identification of additional targets. We focused on human and mouse targets and only retained the most relevant targets with a *P* value of ≤ e−16. To identify potential metabolites of corydine (**1**), we submitted the compound to the GLORY webserver (https://nerdd.zbh.uni-hamburg.de/glory/)^75^. For results validation, corydaline (**2**) was also submitted, and the predicted metabolites were compared to the experimentally identified ones reported in Ji et al*.*^47^ Selected, predicted corydine (**1)** metabolites and metabolites reported by Ji et al.^47^ where the structure could be unequivocally defined, were again subjected to SEA profiling.

### Pharmacology. *Compounds, chemicals and reagents*

Corydine (**1**), corydaline (**2**), bulbocapnine (**3**) and thalictricavine (**4**) were isolated from *Corydalis cava* as previously described^76^. Berberine (**5**) was taken from the inventory of the Institute of Pharmacy/Pharmacognosy of the University of Innsbruck. The natural products **6**–**8** were commercially acquired. Bernumidine (**6**) was obtained from Pharmeks (Moscow, Russia). Intebrimine (**7**) and capnosinine (**8**) were obtained from Interchim (Montluçon, France). The synthetic compound 2-(2,3-dimethoxybenzyl)-6,7-dimethoxy-1,2,3,4-tetrahydroisoquinoline (**9**) was purchased from Ambinter (Orléans, France). The purity of **1** and **2** was determined by LC–MS to be > 98%. Cell culture media and supplements were obtained from Sigma-Aldrich Chemicals (St. Louis, MO). Radioligands [^3^H]DAMGO (50 Ci/mmol), [^3^H]diprenorphine (37 Ci/mmol), [^3^H]U69,593 (60 Ci/mmol) and [^35^S]GTPγS (1,250 Ci/mmol) were purchased from PerkinElmer (Boston, MA). DAMGO, naltrindole, U69,593, unlabeled GTPγS, guanosine diphosphate (GDP), Tris(hydroxymethyl) aminomethane (Tris), and 2-[4-(2-hydroxyethyl)piperazin-1-yl]ethanesulfonic acid (HEPES)were obtained from Sigma-Aldrich Chemicals (St. Louis, MO). Morphine hydrochloride was obtained from Gatt-Koller GmbH (Innsbruck, Austria). Naltrexone hydrochloride was purchased from Siegfried Ltd (Zofingen, Switzerland). PathHunter detection reagents were obtained from DiscoveRx (Birmingham, UK). All other chemicals were of analytical grade and obtained from standard commercial sources. For in vitro assays, morphine and U69,593 were prepared as 1 mM stocks in water. Compounds **1**–**9** and naltrindole were prepared as 1 mM stocks in 0.1% DMSO in water. For in vivo assays, morphine and naltrexone were prepared as 1 mg/ml stocks in sterile physiological saline. Corydine (**1**) and corydaline (**2**) were prepared as 1 mg/ml stocks in 0.1% DMSO in sterile physiological sterile. Stock solutions were further diluted to working concentrations in the appropriate medium.

### ***Cell culture***^26,40^

CHO cells stably expressing human opioid receptors, MOR, DOR or KOR (CHO-hMOR, CHO-hMOR and CHO-hKOR cell lines), were kindly provided by Dr. Lawrence Toll (SRI International, Menlo Park, CA). The CHO-hMOR and CHO-hDOR cell lines were maintained in Dulbecco’s Minimal Essential Medium (DMEM)/Ham’s F-12 medium supplemented with fetal bovine serum (FBS, 10%), penicillin/streptomycin (0.1%), L-glutamine (2 mM) and geneticin (400 µg/ml). The CHO-hKOR cell line was maintained in DMEM supplemented with FBS (10%), penicillin/streptomycin (0.1%), L-glutamine (2 mM) and geneticin (400 µg/ml). U2OS cells stably co-expressing the human MOR and the enzyme acceptor (EA) tagged β-arrestin2 fusion protein (USOS-βarrestin-hMOR-PathHunter cells) (93-0213C3 from DiscoveRx, Birmingham, UK) were cultured in Minimum Essential Medium (MEM) culture medium supplemented with FBS (10%), penicillin/streptomycin (0.1%), L-glutamine (2 mM) and geneticin (500 µg/ml) and hygromycin (250 µg/ml). All cell cultures were maintained in a humidified atmosphere of 95% air and 5% CO_2_.

### Competitive radioligand binding assays

In vitro binding assays were conducted on human opioid receptors stably transfected into CHO cells according to the published procedures^26^. Binding assays were conducted on human opioid receptors stably transfected into CHO cells (CHO-hMOR, CHOhDOR, and CHO-hKOR) according to previously published procedures^26,40^. Cell membranes were prepared as described previously^26^, and stored at − 80 °C until use. Protein content of cell membrane preparations was determined by the method of Bradford using bovine serum albumin as the standard^77^. Cell membranes (15–20 µg) were incubated in 50 mM Tris–HCl buffer (pH 7.4) with 1.1 nM [^3^H]DAMGO (MOR, K_d_ = 1.59 nM), 0.20 nM [^3^H]diprenorphine (DOR, K_d_ = 0.28 nM) or 1.2 nM [^3^H]U69,593 (KOR, K_d_ = 1.47 nM) in a final volume of 1 ml for 60 min at 25 °C. Non-specific binding was determined using 1–10 µM of the unlabeled counterpart of each radioligand. After incubation, reactions were terminated by rapid filtration through Whatman glass GF/C fiber filters. Filters were washed three times with 5 ml of ice-cold 50 mM Tris–HCl buffer (pH 7.4) using a Brandel M24R Cell Harvester (Gaithersburg, MD). Radioactivity retained on the filters was counted by liquid scintillation counting using a Beckman Coulter LS6500 (Beckman Coulter Inc., Fullerton, CA). The inhibitory constant (K_i_) values were calculated from the competition binding curves by nonlinear regression analysis and the Cheng-Prusoff eqaution^78^. All experiments were performed in duplicate, and repeated at least three times with independently prepared samples.

### ***[***^***35***^***S]GTPɣS binding assay***

Binding of [^35^S]GTPγS to membranes from CHO cells stably expressing the human MOR (CHO-hMOR) was conducted according to a previously published procedure^26^. Cell membranes (5–10 µg in 20 mM HEPES, 10 mM MgCl_2_, and 100 mM NaCl, pH 7.4) were incubated with 0.05 nM [^35^S]GTPγS, 10 µM GDP, and test compounds in a final volume of 1 ml for 60 min at 25 °C. Non-specific binding was determined using 10 µM GTPγS, and the basal binding was determined in the absence of test ligand. Samples were filtered over glass Whatman glass GF/B fiber filters and counted as described for binding assays. The increase in [^35^S]GTPγS binding above the basal activity was used to determine potency (EC_50_, in nM) and efficacy (as % stimulation of maximum stimulation with respect to the reference MOR full agonist, DAMGO, which was set as 100%), from concentration–response curves by nonlinear regression analysis. All experiments were performed in duplicate and repeated at least three times with independently prepared samples.

### β-Arrestin2 recruitment assay

The measurement of hMOR stimulated β-arrestin2 recruitment was performed using the PathHunter β-arrestin2 assay (DiscoveRx, Birmingham, UK) according to a previously published procedure^40^^.^ U2OS cells stably co-expressing the human MOR and the enzyme acceptor (EA) tagged β-arrestin2 fusion protein (U2OS-hMOR-βarrestin2 cells) were seeded in cell plating medium into 384-well white plates (Greiner Bio-One, Kremsmünster, Austria) at a density of 5,000 cells in 20 μL per well and maintained for 24 h at 37 °C. After incubation with various concentrations of test compounds in PBS for 90 min at 37 °C, the detection mix was added, and incubation was continued for additional 60 min at room temperature. Chemiluminescence was measured with the PHERAstar FSX Plate Reader (BMG Labtech, Ortenberg, Germany). Potency (EC_50_, in nM) and efficacy (as % stimulation of maximum stimulation with respect to the reference MOR full agonist, DAMGO, which was set as 100%) were determined from concentration–response curves by nonlinear regression analysis. All experiments were performed in duplicate and repeated at least three times with independently prepared samples.

### Animals and drug administration

Male CD1 mice (30–35 g, 7–8 weeks old) were obtained from the Center of Biomodels and Experimental Medicine (CBEM) (Innsbruck, Austria). Mice were group-housed in a temperature-controlled room with a 12 h light/dark cycle and with free access to food and water. All animal studies were conducted in accordance with ethical guidelines and animal welfare standards according to Austrian regulations for animal research and were approved by the Committee of Animal Care of the Austrian Federal Ministry of Science and Research. Test compounds or vehicle were administered by s.c. route in a volume of 10 µL/1 g of body weight.

### Acetic acid-induced writhing test

Writhing was induced in mice by intraperitoneal (i.p.) injection of a 0.6% acetic acid aqueous solution^26^. Drugs or control (vehicle) were s.c. administered, and after 25 min (5 min prior to testing), each animal received i.p. acetic acid solution. Each mouse was placed in individual transparent Plexiglas chambers, and the number of writhes was counted during a 10 min observation period. Antinociceptive activity, as percentage decrease in the number of writhes compared to the control group, was calculated according to the following formula: % inhibition of writhing = 100 × [(C—T)/C], where C is the mean number of writhes in control animals, and T is the number of writhes in drug-treated mice. For the antagonism study, naltrexone (1 mg/kg) was s.c. administered 10 min before the opioid agonist, and writhing as assessed as described above. Each experimental group included five to six animals.

### Data and statistical analysis

Experimental data were analyzed and graphically processed using the GraphPad Prism 5.0 Software (GraphPad Prism Software Inc., San Diego, CA), and are presented as means ± SEM. Data were statistically evaluated using one-way ANOVA with Tukey’s post hoc test or unpaired *t*-test with significance set at *P* < 0.05.

## Supplementary information

Supplementary file1 (PDF 601 kb)
