# Supplementary material for: Identification and characterization of plant-derived alkaloids, corydine and corydaline, as novel mu opioid receptor agonists
Source: Sci Rep. 2020 Aug 14;10:13804. doi: 10.1038/s41598-020-70493-1 (PMC7427800; doi:10.1038/s41598-020-70493-1)
Supplement: Supplementary file 1 — Supplementary file1 (PDF 601 kb) [file 41598_2020_70493_MOESM1_ESM.pdf]

SUPPORTING INFORMATION

Mu Opioid Receptor: Part II. Novel Agonists from Natural Sources and Structural Modeling

Teresa Kaserer,[1] Theresa Steinacher,[1] Roman Kainhofer,[1] Filippo Erli,[1] Sonja Sturm,[2] Birgit Waltenberger,[2]* Daniela Schuster,[1,3]* Mariana Spetea[1]*

[1]Department of Pharmaceutical Chemistry, Institute of Pharmacy and Center for Molecular Biosciences Innsbruck (CMBI), University of Innsbruck, Innrain 80-82, 6020 Innsbruck, Austria

[2]Department of Pharmacognosy, Institute of Pharmacy and Center for Molecular Biosciences Innsbruck (CMBI), University of Innsbruck, Innrain 80-82, 6020 Innsbruck, Austria

[3]Department of Medicinal and Pharmaceutical Chemistry, Institute of Pharmacy, Paracelsus Medical University, Strubergasse 22, 5020, Salzburg, Austria

*Corresponding authors:
Mariana Spetea
E-mail: Mariana.Spetea@uibk.ac.at
Phone: +43-512 507 58277

Daniela Schuster
E-mail: Daniela.Schuster@pmu.ac.at
Phone: +43-662 2420 80610

Birgit Waltenberger
E-mail: Birgit.Waltenberger@uibk.ac.at
Phone: +43-512 507 58420

Table of Contents

Overview on origin and bioactivities of compounds **1-9**……………………………………………..…S3

Figure S1: Binding curves of corydine (**1**) and corydaline (**2**) to the DOR and KOR determined in radioligand binding assays………………………………………………….…………………..……..S5

Table S1: Binding of corydine (**1**) and corydaline (**2**) to the DOR and KOR…………………………S6

Table S2: Predicted off-target activity of corydine (**1**) and corydaline (**2**)……………………….........S7

Figure S2: Structures of predicted corydine (**1**) (**10-15**) and published corydaline (**2**) (**16-22**) metabolites………………………………………………………………………………….………S8

Table S3: Predicted targets for corydine (**1**) (**10-15**) and published corydaline (**2**) (**16-22**) metabolites………………………………………………………………………………..……...S9

References……………………………………………………………………………..……...S12

Overview on origin and bioactivities of compounds 1-9

The aporphine-type alkaloid corydine (**1**) is a natural product known as a constituent of *Corydalis cava*[1] and other plants of the Papaveraceae family, including *Dicranostigma leptopodium*, a medicinal plant with anti-inflammatory, analgesic, antimicrobial and antipyretic effects,[2] and *Dactylicapnos scandens* used in Chinese folk medicine for the treatment of pain.[3] Its isolation has also been reported from plants of other families, including a *Berberis* species, i.e. *Berberis turcomanica* (Berberidaceae).[4] Corydine (**1**) was described to inhibit butyrylcholinesterase[1] and to have antiproliferative,[5] cytochrome P3A4 inhibiting,[6] antimalarial,[7] and antitumor activities.[8] The second aporphine alkaloid, bulbocapnine (**3**), has also been isolated from different plants of the Papaveraceae family, such as *Corydalis cava*[1] and several other *Corydalis* species[9-11] and *Glaucium paucilobum*,[12] which was described to possess analgesic activity,[13] as well as from some other plant families. To the best of our knowledge, it has not been described as a constituent of any *Berberis* species. Bulbocapnine (**3**) has been reported to inhibit acetylcholinesterase,[1] cytochrome P3A4,[6] amyloid-beta protein fibril formation,[14] and dopamine biosynthesis,[15,16] and to exhibit antinociceptive properties.[17]

Corydaline (**2**), a protoberberine alkaloid, has been isolated from different *Corydalis* species (Papaveraceae),[1,18] including *Corydalis yanhusuo*, which is widely used in traditional Chinese medicine for the treatment of pain[19] and whose total alkaloids showed antinociceptive effects in rats.[20] Corydaline (**2**) has also been detected in *Berberis ilicifolia* (Berberidaceae)[21] and some other plants. In addition to other bioactivities, such as nematocidal activity[22], the inhibition of acetylcholinesterase,[1] the inhibition of platelet aggregation,[23] and the increase of gastric emptying,[24], **2** was reported to possess dopamine D_1 receptor antagonistic effects.[25] Thalictricavine (**4**), another protoberberine alkaloid, was described to be a constituent of *Corydalis cava*[26] and *Corydalis tuberosa.*[27] To the best of our knowledge, its presence in any *Berberis* species was not described. Thalictricavine (**4**) was reported to inhibit acetylcholinesterase.[28] The third protoberberine alkaloid, berberine (**5**), is a well-investigated and widely distributed natural product, and one of the main constituents of various *Berberis* species, including the traditional herbal medicine *Berberis vulgaris*, also known as common barberry.[29] It is a major constituent of *Berberis aristata* and was also found in *Berberis crataegina*, both of which have analgesic effects.[30,31] Berberin (**5**) is also found in other Berberidaceae, such as *Mahonia oiwakensis*, which is traditionally used in Taiwan as a medicinal plant for relieving pain, and plant extracts have analgesic and anti-inflammatory effects.[32,33] Moreover, **5** is also present in other plant families. For example, it was identified as a constituent of *Corydalis yanhusuo*[34] and *Corydalis ternata* (Papaveraceae), both of which are used traditionally as pain-relieving medications.[35,36] Berberine (**5**) has shown to exhibit various pharmacological activities, including anti-inflammatory, antioxidant, antihypertensive, antidiabetic, hypolipidemic, antidepressant, anticancer, antimicrobial, and hepatoprotective effects.[37] Furthermore, it was shown to exhibit antinociceptive effects,[30,38-41] and as well as inhibitory effects against morphine-induced

locomotor sensitization and analgesic tolerance.[42] Behavioral studies reported that berberine (**5**) improves intestinal motility and visceral pain in mouse models mimicking diarrhea-predominant irritable bowel syndrome, possibly in an opioid receptor-dependent manner.[39] Bernumidine (**6**) and intebrimine (**7**) are also *Berberis* compounds. However, much less is known about these *N*-benzylisoquinoline alkaloids. The isolation of bernumidine (**6**) has been described only from *Berberis nummularia,*[43] and to the best of our knowledge, no bioactivity was described so far for this alkaloid. Intebrimine (**7**) has been described to be isolated only from *Berberis integerrima.*[43] It showed weak antiproliferative activity on malignant human cells.[44]

The chemical structure of capnosinine (**8**) is similar to that of intebrimine (**7**), with the exception that it comprises a hydroxy group instead of a methoxy group in position 3'. However, to date, **8** was not described as a *Berberis* compound. Instead, its isolation was described from *Ceratocapnos heterocarpa*, belonging to the Paperaceae plant family.[45] The substitution pattern of the only synthetic compound, **9**, is also similar to that of natural product intebrimine (**7**). The only difference between the structures of **7** and **9** is that the two methoxy functions in the benzyl group are in positions 2' and 3' in **9**, while they are in positions 3' and 4' in **7**.

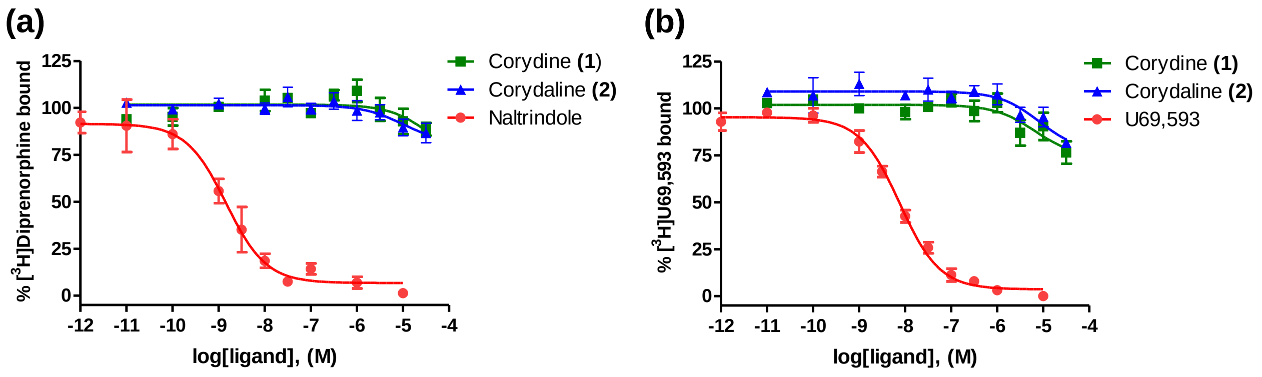

Figure S1. Binding curves of corydine (**1**) and corydaline (**2**) to the DOR and KOR determined in radioligand binding assays. (**a**) Concentration-dependent inhibition by corydine (**1**), corydaline (**2**) and naltrindole (reference DOR ligand) of [^{3}H]diprenorphine binding to membranes from CHO cells stably expressing the human DOR. (**b**) Concentration-dependent inhibition by corydine (**1**), corydaline (**2**) and U69,593 (reference KOR ligand) of [^{3}H]U69,593 binding to membranes from CHO cells stably expressing the human KOR. Values are means ± SEM (n = 3 independent experiments performed in duplicate).

Table S1. Binding of Corydine (**1**) and Corydaline (**2**) to the DOR and KOR

**Compound**	**K_i /K_d(nM)[a]**	
	**DOR**	**KOR**
Corydine (**1**)	-[b]	-[b]
Corydaline (**2**)	-[b]	-[b]
Naltrindole	0.64 ± 0.17	n.d.
U69,593	n.d.	1.54 ± 0.05

[a]Determined in competitive radioligand binding assays using CHO cell membranes stably expressing the human DOR or the human KOR. Naltrindole was used as reference DOR ligand, and U69,593 was used as reference KOR ligand. [b]- denotes no substantial inhibitory activity in the concentration range tested to permit a formal calculation of K_i values (see Figure S1). n.d. denotes not determined. Values are means ± SEM (n = 3 independent experiments performed in duplicate).

Table S2. Predicted Off-Target Activity of Corydine (**1**) and Corydaline (**2**)[a]

**Compound**	**Target (species)**	***P* value**
Corydine (**1**)	D(1A) dopamine receptor (mouse)	3.654e-56
	D(1A) dopamine receptor (human)	3.166e-49
	Tyrosine 3-monooxygenase (human)	1.905e-37
	Peptidyl-prolyl cis-trans isomerase FKBP1A (human)	7.312e-32
	D(2) dopamine receptor (human)	7.829e-21
	Protein tyrosine phosphatase receptor type C-associated protein (human)	4.690e-20
	5-hydroxytryptamine receptor 1A (human)	4.481e-20
	Neuronal acetylcholine receptor subunit beta-4 (mouse)	8.912e-20
	D(1B) dopamine receptor (human)	4.073e-19
	Neuronal acetylcholine receptor subunit beta-2 (mouse)	1.081e-18
	Neuronal acetylcholine receptor subunit alpha-2 (human)	1.407e-17
Corydaline (**2**)	D(1A) dopamine receptor (human)	6.203e-38
	Potassium/sodium hyperpolarization-activated cyclic nucleotide-gated channel 2 (mouse)	1.266e-31
	Multidrug resistance protein 1B (mouse)	1.208e-23
	D(1B) dopamine receptor (human)	7.461e-21
	Neuronal acetylcholine receptor subunit beta-4 (mouse)	4.599e-19
	Neuronal acetylcholine receptor subunit beta-2 (mouse)	5.083e-18
	Calcium/calmodulin-dependent 3’,5’-cyclic nucleotide phosphodiesterase 1B (human)	1.325e-17
	Multidrug resistance protein 1 (human)	1.110e-16

[a]*In silico* profiling tool Similarity Ensemble Approach (SEA; http://sea.bkslab.org/)[46] was used.

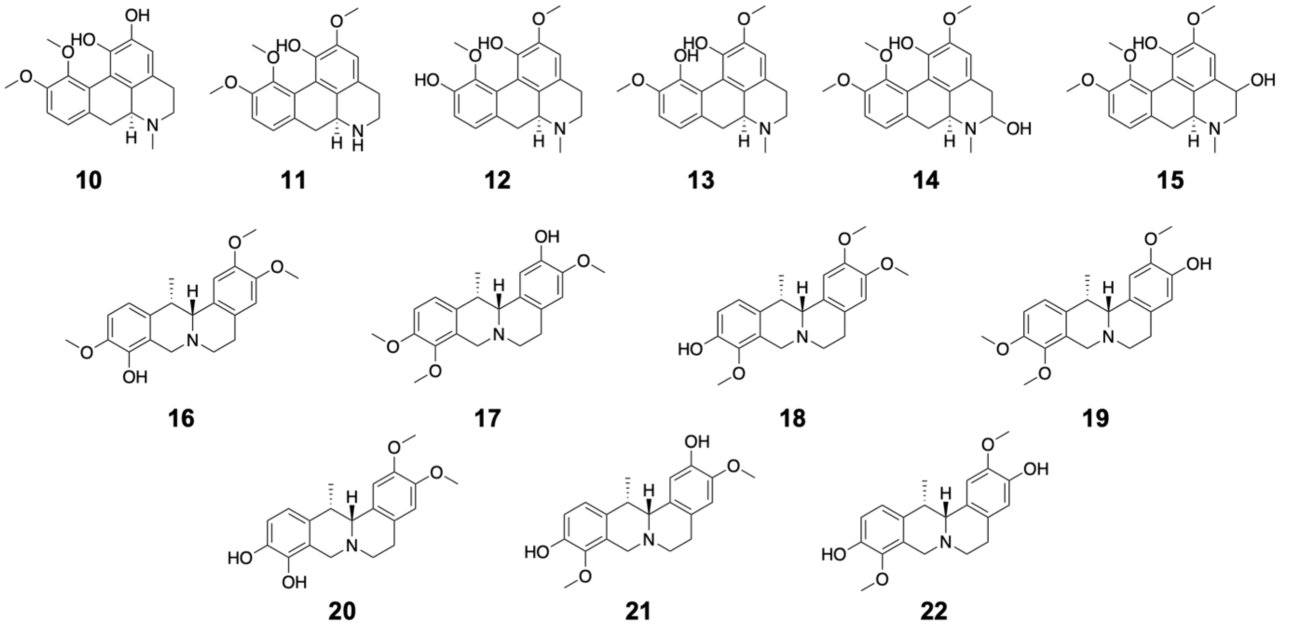

Figure S2. Structures of Predicted Corydine (**1**) (**10**-**15**) and Published[47] Corydaline (**2**) (**16**-**22**) Metabolites.

Table S3. Predicted Target Activity of Corydine (**1**) (**10**-**15**) and Published[47] Corydaline (**2**) (**16**-**22**) Metabolites[a]

Metabolite	Target (species)	*P* value
**10**	D(1A) dopamine receptor (mouse)	5.406e-61
	D(1A) dopamine receptor (human)	2.007e-49
	Tyrosine 3-monooxygenase (human)	2.935e-41
	Peptidyl-prolyl cis-trans isomerase FKBP1A (human)	1.334e-30
	Neuronal acetylcholine receptor subunit beta-4 (mouse)	9.356e-21
	D(2) dopamine receptor (human)	4.613e-20
	Neuronal acetylcholine receptor subunit beta-2 (mouse)	1.289e-19
	Protein tyrosine phosphatase receptor type C-associated protein (human)	1.225e-18
	5-hydroxytryptamine receptor 1A (human)	1.030e-18
	D(1B) dopamine receptor (human)	1.250e-17
**11**	Protein tyrosine phosphatase receptor type C-associated protein (human)	8.446e-33
**12**	D(1A) dopamine receptor (mouse)	5.116e-62
	D(1A) dopamine receptor (human)	6.953e-53
	Tyrosine 3-monooxygenase (human)	6.549e-42
	Peptidyl-prolyl cis-trans isomerase FKBP1A (human)	7.312e-32
	D(2) dopamine receptor (human)	1.226e-21
	D(1B) dopamine receptor (human)	2.922e-21
	Neuronal acetylcholine receptor subunit beta-4 (mouse)	8.784e-21
	5-hydroxytryptamine receptor 1A (human)	3.452e-20
	Neuronal acetylcholine receptor subunit beta-2 (mouse)	1.214e-19
	Protein tyrosine phosphatase receptor type C-associated protein (human)	6.333e-19
	Neuronal acetylcholine receptor subunit alpha-2 (human)	1.407e-17
	Dihydropteridine reductase (human)	5.551e-16
**13**	D(1A) dopamine receptor (mouse)	4.776e-58
	D(1A) dopamine receptor (human)	1.168e-50
	Tyrosine 3-monooxygenase (human)	1.258e-38
	Peptidyl-prolyl cis-trans isomerase FKBP1A (human)	7.612e-32
	D(2) dopamine receptor (human)	1.191e-21
	5-hydroxytryptamine receptor 1A (human)	3.886e-21
	D(1B) dopamine receptor (human)	6.498e-21
	Neuronal acetylcholine receptor subunit beta-4 (mouse)	1.876e-20
	Neuronal acetylcholine receptor subunit beta-2 (mouse)	2.484e-19
	Protein tyrosine phosphatase receptor type C-associated protein (human)	2.776e-17
	Neuronal acetylcholine receptor subunit alpha-2 (human)	2.941e-17
**14**	D(1A) dopamine receptor (mouse)	1.983e-40
	Tyrosine 3-monooxygenase (human)	8.367e-31
	D(1A) dopamine receptor (human)	4.498e-30
	Protein tyrosine phosphatase receptor type C-associated protein (human	1.450e-17
**15**	D(1A) dopamine receptor (mouse)	7.020e-44
	Tyrosine 3-monooxygenase (human)	5.998e-30

	D(1A) dopamine receptor (human)<br>Calcium/calmodulin-dependent 3',5'-cyclic nucleotide phosphodiesterase 1B (human)<br>Protein tyrosine phosphatase receptor type C-associated protein (human)	5.591e-29<br>5.887e-21<br><br>4.441e-16
**16**	D(1A) dopamine receptor (human)<br>Potassium/sodium hyperpolarization-activated cyclic nucleotide-gated channel 2 (mouse)<br>Neuronal acetylcholine receptor subunit beta-4 (mouse)<br>Neuronal acetylcholine receptor subunit beta-2 (mouse)<br>D(1B) dopamine receptor (human)<br>Muscarinic acetylcholine receptor M4 (human)<br>D(1A) dopamine receptor (mouse)<br>Multidrug resistance protein 1 (human)	1.413e-39<br>3.458e-31<br><br>4.790e-24<br>1.013e-22<br>1.859e-19<br>3.142e-18<br>5.823e-18<br>1.110e-16
**17**	D(1A) dopamine receptor (human)<br>D(1B) dopamine receptor (human)<br>Tubulin beta-1 chain (human)<br>Neuronal acetylcholine receptor subunit beta-4 (mouse)<br>Neuronal acetylcholine receptor subunit beta-2 (mouse)<br>Tubulin beta-3 chain (human)	7.053e-47<br>2.457e-25<br>4.623e-24<br>2.013e-21<br>3.025e-20<br>8.882e-16
**18**	D(1A) dopamine receptor (human)<br>D(1A) dopamine receptor (mouse)<br>Tyrosine 3-monooxygenase (human)<br>Neuronal acetylcholine receptor subunit beta-4 (mouse)<br>Neuronal acetylcholine receptor subunit beta-2 (mouse)<br>D(1B) dopamine receptor (human)<br>Multidrug resistance protein 1B (mouse)	8.018e-43<br>3.754e-31<br>2.272e-30<br>1.494e-24<br>3.374e-23<br>4.688e-22<br>2.220e-16
**19**	D(1A) dopamine receptor (human)<br>Neuronal acetylcholine receptor subunit beta-4 (mouse)<br>Neuronal acetylcholine receptor subunit beta-2 (mouse)<br>D(1B) dopamine receptor (human)<br>Tubulin beta-1 chain (human)<br>Matrix metalloproteinase-26 (human)<br>Tubulin beta-3 chain (human)	1.209e-42<br>9.804e-22<br>1.534e-20<br>4.463e-19<br>2.330e-19<br>6.661e-16<br>8.882e-16
**20**	D(1A) dopamine receptor (human)<br>D(1A) dopamine receptor (mouse)<br>Tyrosine 3-monooxygenase (human)<br>Neuronal acetylcholine receptor subunit beta-4 (mouse)<br>Neuronal acetylcholine receptor subunit beta-2 (mouse)<br>D(1B) dopamine receptor (human)<br>Muscarinic acetylcholine receptor M4 (human)<br>Potassium/sodium hyperpolarization-activated cyclic nucleotide-gated channel 2 (mouse)	5.147e-47<br>4.140e-40<br>8.525e-35<br>1.282e-24<br>2.922e-23<br>1.623e-19<br>3.576e-17<br>9.992e-16
**21**	D(1A) dopamine receptor (human)<br>Tyrosine 3-monooxygenase (human)<br>D(1A) dopamine receptor (mouse)<br>D(1B) dopamine receptor (human)<br>Neuronal acetylcholine receptor subunit beta-4 (mouse)<br>Neuronal acetylcholine receptor subunit beta-2 (mouse)	4.498e-47<br>5.998e-30<br>1.267e-26<br>5.598e-25<br>2.013e-21<br>3.025e-20

	Dihydropteridine reductase (human)	8.882e-16
**22**	D(1A) dopamine receptor (human)<br>Tyrosine 3-monooxygenase (human)<br>D(1A) dopamine receptor (mouse)<br>Neuronal acetylcholine receptor subunit beta-4 (mouse)<br>Neuronal acetylcholine receptor subunit beta-2 (mouse)<br>D(1B) dopamine receptor (human)<br>Matrix metalloproteinase-26 (human)<br>Dihydropteridine reductase (human)	3.744e-42<br>5.998e-30<br>1.267e-26<br>9.804e-22<br>1.534e-20<br>9.356e-18<br>6.661e-16<br>8.882e-16

[a]*In silico* profiling tool Similarity Ensemble Approach (SEA; http://sea.bkslab.org/)[46] was used.

References

[1] Adsersen, A., Kjoelbye, A., Dall, O., Jaeger, A. K. Acetylcholinesterase and butyrylcholinesterase inhibitory compounds from *Corydalis cava* Schweigg. & Kort. *J Ethnopharmacol* **113**, 179-182 (2007).

[2] Chang, H.-J., Wang, H.-H., Ma, K.-E. Study on chemical constituents of *Dicranostigma leptopodium* (Maxim.) Fedde. *Yaoxue Tongbao* **16**, 52 (1981).

[3] Wang, X., Dong, H., Yang, B., Liu, D., Duan, W., Huang, L. Preparative isolation of alkaloids from *Dactylicapnos scandens* using pH-zone-refining counter-current chromatography by changing the length of the separation column. *J Chromatogr B Analyt Technol Biomed Life Sci* **879**, 3767-3770 (2011).

[4] Karimov, A., Levkovich, M. G., Abdullaev, N. D., Shakirov, R. Berberis alkaloids. XXIII. Structure of turcberine. *Khim Prirodn Soedin* **1**, 77-81 (1993).

[5] Rong, L., Hu, D., Wang, W., Zhao, R., Xu, X., Jing, W. Alkaloids from root tubers of *Stephania kwangsiensis* H.S.Lo and their effects on proliferation and apoptosis of lung NCI-H446 cells. *Biomed Res (Aligarh, India)* **27**, 893-896 (2016).

[6] Salminen, K. A., Meyer, A., Jerabkova, L., Korhonen, L. E., Rahnasto, M., Juvonen, R. O., Imming, P., Raunio, H. Inhibition of human drug metabolizing cytochrome P450 enzymes by plant isoquinoline alkaloids. *Phytomedicine* **18**, 533-538 (2011).

[7] Weniger, B., Aragon, R., Deharo, E., Bastida, J., Codina, C., Lobstein, A., Anton, R. Antimalarial constituents from *Guatteria amplifolia*. *Pharmazie* **55**, 867-868 (2000).

[8] Kondo, Y., Imai, Y., Hojo, H., Endo, T., Nozoe, S. Suppression of tumor cell growth and mitogen response by aporphine alkaloids, dicentrine, glaucine, corydine, and apomorphine. *J Pharmacobiodyn* **13**, 426-431 (1990).

[9] Naruto, S., Namba, K., Kaneko, H. Constituents of Corydalis species. IX. Alkaloids from several tuberous Corydalis species. *Phytochemistry* **11**, 2642-2643 (1972).

[10] Kiryakov, Kh. G., Israilov, I. A., Yunusov, S. Y. Alkaloids of *Corydalis marschalliana*. *Khim Prirodn Soedin* **3**, 411 (1974).

[11] Gheorghiu, A., Ionescu-Matiu, E., Manuchian, M. [Alkaloids of] *Corydalis solida*. *Ann Pharm Fr* **20**, 468-477 (1962).

[12] Shafiee, A., Morteza-Semnani, K. Crabbine and other alkaloids from the aerial parts of *Glaucium paucilobum*. *Planta Med* **64**, 680 (1998).

[13] Morteza-Semnani, K., Mahmoudi, M., Heidar, M. R. Analgesic activity of the methanolic extract and total alkaloids of *Glaucium paucilobum*. *Methods Find Exp Clin Pharmacol* **28**, 151-155 (2006).

[14] Lashuel, H. A., Hartley, D. M., Balakhaneh, D., Aggarwal, A., Teichberg, S., Callaway, D. J. New class of inhibitors of amyloid-beta fibril formation. Implications for the mechanism of pathogenesis in Alzheimer's disease. *J Biol Chem* **277**, 42881-42890 (2002).

[15] Zhang, Y. H., Shin, J. S., Lee, S. S., Kim, S. H., Lee, M. K. Inhibition of tyrosine hydroxylase by bulbocapnine. *Planta Med* **63**, 362-363 (1997).

[16] Shin, J. S., Kim, K. T., Lee, M. K. Inhibitory Effects of bulbocapnine on dopamine biosynthesis in PC12 Cells. *Neurosci Lett* **244**, 161-164 (1998).

[17] Zetler, G. Neuroleptic-like, anticonvulsant and antinociceptive effects of aporphine alkaloids: bulbocapnine, corytuberine, boldine and glaucine. *Arch Int Pharmacodyn Ther* **296**, 255-281 (1988).

[18] Han, J. W., Jang, K. S., Choi, Y. H., Kim, H., Choi, G. J., Shim, S. H., Kim, H., Choi, G. J. In vivo disease control efficacy of isoquinoline alkaloids isolated from *Corydalis ternata* against wheat leaf rust and pepper anthracnose. *J Microbiol Biotechnol* **28**, 262-266 (2018).

[19] Ma, Z.-Z., Xu, W., Jensen, N. H., Roth, B. L., Liu-Chen, L.-Y., Lee, D. Y. W. Isoquinoline alkaloids isolated from *Corydalis yanhusuo* and their binding affinities at the dopamine D1 receptor. *Molecules* **13**, 2303-2312 (2008).

[20] Wang, C., Wang, S., Fan, G., Zou, H. Screening of antinociceptive components in *Corydalis yanhusuo* W.T. Wang by comprehensive two-dimensional liquid chromatography/tandem mass spectrometry. *Anal Bioanal Chem* **396**, 1731-1740 (2010).

[21] Ruiz, A.; Zapata, M.; Sabando, C.; Bustamante, L.; von Baer, D.; Vergara, C.; Mardones, C. Flavonols, alkaloids, and antioxidant capacity of edible wild Berberis species from Patagonia. *J Agric Food Chem* **62**, 12407-12417 (2014).

[22] Satou, T., Koga, M., Matsuhashi, R., Koike, K., Tada, I., Nikaido, T. Assay of nematocidal activity of isoquinoline alkaloids using third-stage larvae of *Strongyloides ratti* and *S. venezuelensis*. *Vet Parasitol* **104**, 131-138 (2002).

[23] Zhang, Q., Chen, C., Wang, F.-Q., Li, C. H., Zhang, Q. H., Hu, Y. J., Xia, Z. N., Yang, F. Q. Simultaneous screening and analysis of antiplatelet aggregation active alkaloids from *Rhizoma Corydalis*. *Pharm Biol* **54**, 3113-3120 (2016).

[24] Lee, T. H., Son, M., Kim, S. Y. Effects of corydaline from *Corydalis tuber* on gastric motor function in an animal model. *Biol Pharm Bull* **33**, 958-962 (2010).

[25] Wu, L., Zhang, W., Qiu, X., Wang, C., Liu, Y., Wang, Z., Yu, Y., Ye, R. D., Zhang, Y. Identification of alkaloids from *Corydalis yanhusuo* W. T. Wang as dopamine D1 receptor antagonists by using CRE-luciferase reporter gene assay. *Molecules* **23**, 2585 (2018).

[26] Slavik, J., Slavikova, L. Alkaloids of the Papaveraceae. LXVII. Alkaloids from *Corydalis cava* (L.) Schw. et Koerte. *Collection Czechoslovak Chem Commun* **44**, 2261-2274 (1979).

[27] Manske, R. H. F. The alkaloids of fumariaceous plants. XLIX. Thalictricavine, a new alkaloid from *Corydalis tuberosa* DC. *J Am Chem Soc* **75,** 4928-4929 (1953).

[28] Chlebek, J., Korabecny, J., Dolezal, R., Stepankova, S., Perez, D. I., Hošťálková A., Opletal, L., Cahlikova, L., Macakova, K., Kucera, T., Hrabinová, M., Jun, D. In vitro and in silico acetylcholinesterase inhibitory activity of thalictricavine and canadine and their predicted penetration across the blood-brain barrier. *Molecules* **24**, 1340 (2019).

[29] Mokhber-Dezfuli, N., Saeidnia, S., Gohari, A. R., Kurepaz-Mahmoodabadi, M. Phytochemistry and pharmacology of Berberis species. *Pharmacogn Rev* **8**, 8-15 (2014).

[30] Shahid, M., Rahim, T., Shahzad, A., Tajuddin, T., Latif, A., Fatma, T., Rashid, M., Raza, A., Mustafa, S. Ethnobotanical studies on *Berberis aristata* DC. root extracts. *Afr J Biotechnol* **8**, 556-563 (2009).

[31] Yesilada, E., Kupeli E. *Berberis crataegina* DC. root exhibits potent anti-inflammatory, analgesic and febrifuge effects in mice and rats. *J Ethnopharmacol* **79**, 237-248 (2002).

[32] Chao, J., Lu, T.-C., Liao, J.-W., Huang, T.-H., Lee, M.-S., Cheng, H.-Y., Ho, L.-K., Kuo, C.-L., Peng, W.-H. Analgesic and anti-inflammatory activities of ethanol root extract of *Mahonia oiwakensis* in mice. *J Ethnopharmacol* **125**, 297-303 (2009).

[33] Chao, J., Liao, J.-W., Peng, W.-H., Lee, M.-S., Pao, L.-H., Cheng, H.-Y. Antioxidant, analgesic, anti-inflammatory, and hepatoprotective effects of the ethanol extract of *Mahonia oiwakensis* stem. *Int J Mol Sci* **14**, 2928-2945 (2013).

[34] Gao, J.-L., Shi, J.-M., Lee, S. M.-Y., Zhang, Q.-W., Wang, Y.-T. Angiogenic pathway inhibition of *Corydalis yanhusuo* and berberine in human umbilical vein endothelial cells. *Oncol Res* **17**, 519-526 (2009).

[35] Lee, H. Y., Kim, C. W. Isolation and quantitative determination of berberine and coptisine from tubers of *Corydalis ternata*. *Saengyak Hakhoechi* **30**, 332-334 (1999).

[36] Kim, Y. J., Lim, H.-S., Kim, Y., Lee, J., Kim, B.-Y., Jeong, S.-J. Neuroprotective effect of *Corydalis ternata* extract and its phytochemical quantitative analysis. *Chem Pharm Bull* **65**, 826-832 (2017).

[37] Singh, A., Duggal, S., Kaur, N., Singh J. Berberine: alkaloid with wide spectrum of pharmacological activities. *J Nat Prod* **3**, 64-75 (2010).

[38] Zhang, M., Shen, Y., Tang, C. Anti-inflammatory and analgesic actions of berberine. *Tianran Chanwu Yanjiu Yu Kaifa Kaifa* **2**, 49-53 (1990).

[39] Chen, C., Lu, M., Pan, Q., Fichna, J., Zheng, L., Wang, K., Yu, Z., Li, Y., Li, K., Song, A., Liu, Z., Song, Z., Kreis, M. Berberine improves intestinal motility and visceral pain in the mouse models mimicking diarrhea-predominant irritable bowel syndrome (IBS-D) symptoms in an opioid-receptor dependent manner. *PLoS One* **10**, e0145556 (2015).

[40] Tang, Q. L., Lai, M. L., Zhong, Y. F., Wang, A. M., Su, J. K., Zhang, M. Q. Antinociceptive effect of berberine on visceral hypersensitivity in rats. *World J Gastroenterol* **19**, 4582-4589 (2013).

[41] Küpeli, E., Koşar, M., Yeşilada, E., Hüsnü, K., Başer, C. A comparative study on the anti-inflammatory, antinociceptive and antipyretic effects of isoquinoline alkaloids from the roots of Turkish Berberis species *Life Sci* **72**, 645-657 (2002).

[42] Yoo, J. H., Yang, E. M., Cho, J. H., Lee, J. H., Jeong, S. M., Nah, S. Y., Kim, H. C., Kim, K. W., Kim, S. H., Lee, S. Y., Jang, C. G. Inhibitory effects of berberine against morphine-induced locomotor sensitization and analgesic tolerance in mice. *Neuroscience* **142**, 953-961 (2006).

[43] Karimov, A., Vinogradova, V. I., Shakirov, R. Berberis alkaloids. XXII. Intebrinine and intebrimine, new alkaloids from *Berberis integerrima*. *Khim Prirodn Soedin* **1**, 70-73 (1993).

[44] Ho, S. S., Go, M. L. Restraining the flexibility of the central linker in terameprocol results in constrained analogs with improved growth inhibitory activity. *Bioorg Med Chem Lett* **23**, 6127-6133 (2013).

[45] Suau, R. I., Silva, M. V., Ruiz, I., Valpuesta, M. *N*-Benzylisoquinoline alkaloids from *Ceratocapnos heterocarpa*. *Phytochemistry* **36**, 241-243 (1994).

[46] Keiser, M. J., Roth B. L., Armbruster, B. N., Ernsberger. P., Irwin, J. J., Shoichet, B. K. Relating protein pharmacology y ligand chemistry. *Nat Biotech* **25**, 197-206 (2007).

[47] Ji, H. Y., Lee, H., Kim, J. H., Kim, K. H., Lee, K. R., Shim, H. J., Son, M., Lee, H. S. In vitro metabolism of corydaline in human liver microsomes and hepatocytes using liquid chromatography-ion trap mass spectrometry. *J Sep Sci* **35**, 1102-1109 (2012).
